# p16 Expression Is Lost in Severely Atypical Cellular Blue Nevi and Melanoma Compared to Conventional, Mildly, and Moderately Atypical Cellular Blue Nevi

**DOI:** 10.1155/2014/348417

**Published:** 2014-01-22

**Authors:** Laura M. Chang, David S. Cassarino

**Affiliations:** ^1^Department of Dermatology, Southern California Kaiser Permanente, Los Angeles Medical Center (LAMC), Kaiser Permanente, 4867 W Sunset Boulevard, Los Angeles, CA 90027-5969, USA; ^2^Department of Pathology, Southern California Kaiser Permanente, Los Angeles Medical Center (LAMC), Kaiser Permanente, 4867 W Sunset Boulevard, Los Angeles, CA 90027-5969, USA

## Abstract

*Background*. Significant decreases in p16 expression have been shown to occur in melanoma compared to Spitz tumors, and loss of p16 staining has been found to correlate with melanoma tumor progression. However, comparison of p16 between atypical cellular blue nevi (CBN) and melanoma has not been reported previously. *Methods*. p16 immunohistochemical staining was evaluated in 14 atypical CBN, 8 conventional and atypical melanocytic nevi, and 16 melanomas, including 4 malignant CBN. p16 staining intensity was graded on a scale of 0–3 and the percentage of melanocytes stained with p16 was determined. *Results*. p16 staining was significantly higher in all CBN as a group when compared to melanomas (*P* = 0.001) and malignant CBN (*P* = 0.00008). Higher p16 expression was also seen in mildly (*P* = 0.0002) and moderately atypical (*P* = 0.02), but not severely atypical, CBN compared to melanomas. *Conclusions*. p16 immunohistochemical expression is higher in mildly and moderately atypical CBN compared to severely atypical CBN and melanomas. In conjunction with additional markers and histology, p16 staining may be useful in confirming the benign nature of these tumors, but is not useful in distinguishing severely atypical CBN from malignant cases, consistent with the overlapping histologic features between these tumors.

## 1. Introduction

Distinguishing atypical and unusual variants of melanocytic nevi from melanoma by routine histologic examination can be difficult in some cases [1–3]. In particular, differentiating atypical cellular blue nevi (CBN) from melanoma (including melanoma arising in or mimicking a cellular blue nevus, the so-called “malignant cellular blue nevus”) can pose a significant diagnostic problem [1], and it has been shown that even experienced dermatopathologists often disagree in differentiating CBN, especially atypical CBN, from melanoma [4]. Like melanoma, CBN often lack maturation, can have dermal mitotic figures, extend deeply in the dermis, and may have perineural and even intralymphatic involvement [5].

Immunohistochemistry is a useful tool in the diagnosis of some cases of melanoma, and markers such as S-100, HMB-45, Melan-A, MITF, and the proliferation marker Ki-67 are often used. Ki-67, in particular, has been found useful to distinguish benign from malignant melanocytic lesions [6], but additional markers would clearly be beneficial. p16 is one of the proteins that regulates the G1/S checkpoint of the cell cycle, and it is the product of the tumor suppressor gene *CDKN2* [7]. Since loss of p16 expression has been documented to occur in melanoma [8], p16 may be a potential helpful marker in differentiating atypical melanocytic nevi from melanoma. p16 has been shown to be decreased or absent in melanoma compared to benign melanocytic nevi [9–13], including congenital melanocytic nevi [14]. This loss of p16 staining in melanoma compared to benign nevi has also been found to occur in noncutaneous sites such as the oral mucosa and conjunctiva [15, 16]. Furthermore, p16 has also been shown to be helpful for discriminating special types of nevi, such as Spitz nevi, from melanoma [17, 18], including differentiating desmoplastic Spitz nevi from desmoplastic melanoma [19].

Examination of p16 immunohistochemical staining in CBN and in particular atypical CBN, which are difficult to differentiate from melanoma arising in or mimicking a CBN, has not yet been reported. In this study, we analyzed p16 expression in benign, mildly, moderately, and severely atypical CBN and compared these findings with cases of melanoma, malignant CBN, conventional benign, and atypical melanocytic nevi. We also examined Ki-67 in these lesions in order to compare this well-known marker to p16 to further evaluate the usefulness of p16 as an adjunct in distinguishing benign and atypical CBN from melanoma.

## 2. Materials and Methods

Paraffin-embedded, formalin-fixed blocks were obtained from 15 CBN, 8 conventional and atypical melanocytic nevi, 12 melanomas, and 4 malignant CBN from the Kaiser Permanente Southern California pathology files. This study was approved by the Kaiser Permanente Southern California institutional review board. Among the CBN, one was conventional CBN, five were mildly atypical, four were moderately atypical, and five were severely atypical. The histologic grading was based on architectural features (including increased cellularity, dense, sheet-like areas, and large size of severely atypical lesions) and cytologic features (including the degree of nuclear enlargement, hyperchromasia, pleomorphism, and prominent nucleoli, as well as the presence of mitotic figures [up to 1/10 hpf in mildly atypical CBN, up to 2-3/10 hpf in moderately atypical CBN, and greater than 3/10 hpf and/or atypical mitoses in severely atypical nevi]). The conventional benign and atypical melanocytic nevi consisted of compound congenital nevi and atypical nevi ranging in atypia from mild to severe. The melanomas included a melanoma in situ, seven superficial spreading melanomas, two spitzoid melanomas, a nevoid melanoma, and a melanoma metastasis. In addition, we examined four frankly malignant cellular blue nevi (three primary and one recurrent). The depth of the invasive melanomas ranged from 0.3 mm to 5.0 mm.

Immunohistochemical staining for p16, HMB-45 (only select cases), and Ki-67 was performed on 5 *μ*m sections of the paraffin-embedded tissues. p16 staining was performed on all the specimens, Ki-67 was performed on all but one severely atypical cellular blue nevus, six of eight conventional nevi, and seven of 12 melanomas. HMB-45 was performed on three moderately/severely atypical CBN, four of eight conventional nevi, and six of 12 melanomas. p16 immunohistochemical staining was performed using the CINtec Histology Kit (Roche, Tucson, AZ) which utilizes the p16 antibody clone E6H4.

p16 (nuclear and cytoplasmic) and HMB-45 (cytoplasmic) staining intensity were graded on a scale of 0–3 (1: weak, 2: moderate, and 3: strong), and the percentage of melanocytes positive for p16, HMB-45, and Ki-67 was determined. Each tumor was independently evaluated by two reviewers, and the results were averaged.

Statistical significance was calculated using the analysis of variance (ANOVA) test and the two-tailed, two sample unequal variance *t*-test. ANOVA testing showed that p16 and Ki-67 staining results were statistically significant for the comparisons made among the multiple tumor categories.

## 3. Results

**Figure 1:**
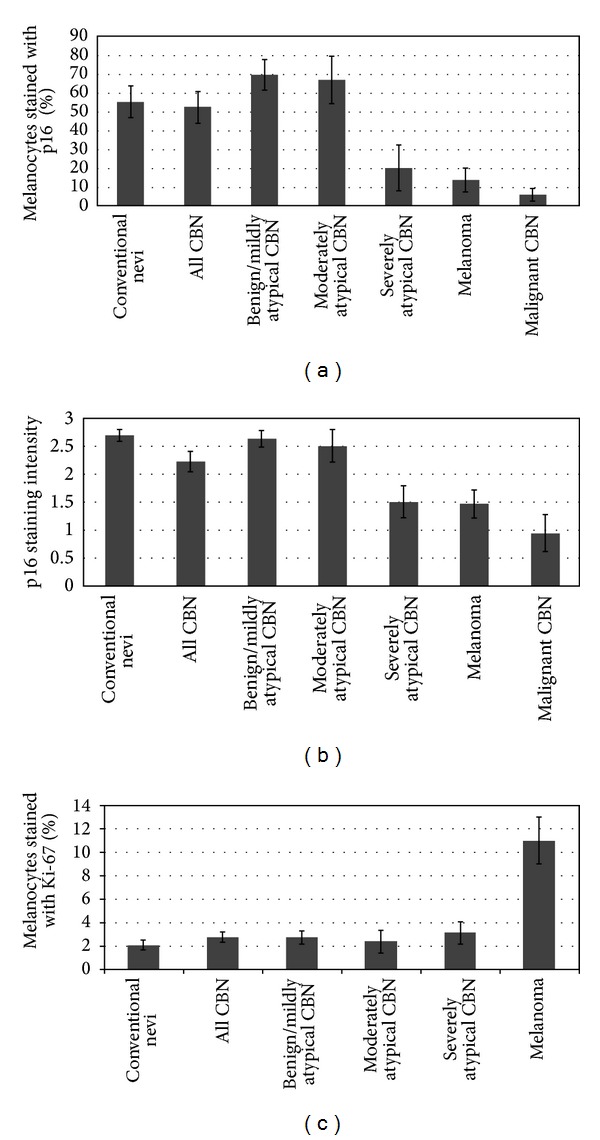
p16 staining intensity (a) and percentage of melanocytes stained with p16 (b) and Ki-67 (c) in conventional benign and atypical nevi, CBN, melanoma, and malignant CBN. (a, b) p16 staining was higher in all CBN when compared to melanoma (*P* = 0.02 and *P* = 0.001) and malignant CBN (*P* = 0.02 and *P* = 0.00008). Upon subclassifying the CBN based on degree of atypia, a significant increase in p16 staining in benign/mildly atypical CBN (*P* = 0.0002) and moderately atypical CBN (*P* = 0.02) but not severely atypical CBN was found compared to melanoma. Similar results were obtained when comparing these classes of CBN with malignant CBN. Compared to conventional nevi, benign/mildly and moderately atypical CBN had similar p16 staining, but a significant decrease in p16 was found in severely atypical CBN (*P* = 0.04). (c) Ki-67 was significantly lower in CBN of all levels of atypia compared to melanoma (*P* < 0.03).

Patient clinical characteristics and follow-up data are summarized in Table 1. When comparing p16 expression in all CBN with melanoma, we found a statistically significant higher level of p16 staining intensity (2.22 ± 0.18 versus 1.46 ± 0.25, *P* = 0.02) and percentage of melanocytes positive with p16 (52.4 ± 8.33 versus 13.92 ± 6.32, *P* = 0.001) in the CBN (Figures 1(a) and 1(b), Table 2). p16 staining was generally strong, with both nuclear and cytoplasmic staining, in the CBN. A similar, and in some instances an even stronger difference between CBN and malignant CBN was found for p16 staining intensity (2.22 ± 0.18 versus 0.94 ± 0.33, *P* = 0.02) and percentage of positive melanocytes staining with p16 (52.4 ± 8.33 versus 6 ± 3.24, *P* = 0.00008) (Figures 1(a) and 1(b), Table 3). Although 10 of 12 melanomas displayed only very low levels of p16 staining, with 0–12.5% of melanocytes staining for p16 (and typically showing only weak cytoplasmic staining), one superficial spreading melanoma did not show significant loss of p16 (with 80% of melanocytes staining for p16, and a staining intensity of 3), and a nevoid melanoma had moderate levels of p16 staining (with 25% of melanocytes staining for p16, and a staining intensity of 2.75).

When the CBN were compared to the conventional and atypical melanocytic nevi, a small but statistically significant decrease in p16 staining intensity was found (*P* = 0.04), but not in percentage of melanocytes stained for p16 (Figures 1(a) and 1(b), Table 4). As expected, Ki-67 was significantly lower in CBN compared to melanoma (2.75 ± 0.42 versus 11 ± 1.98, *P* = 0.02) (Figure 1(c), Table 2), but no significant difference was found between CBN and conventional nevi (Figure 1(c), Table 4). HMB-45 had strong staining intensity in three moderately to severely atypical CBN and six melanomas, but the average percentage of melanocytes stained with HMB-45 was higher among the melanomas (data not shown).

**Figure 2:**
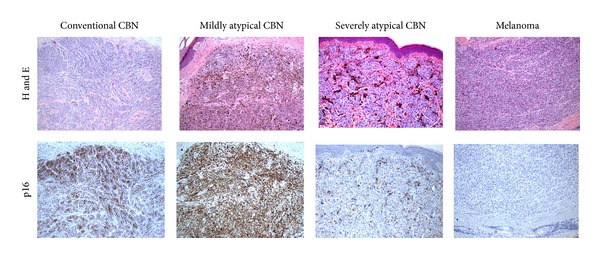
H and E and p16 staining of representative conventional CBN, mildly atypical CBN, severely atypical CBN, and a melanoma (metastatic) mimicking CBN. p16 staining intensity: benign CBN = 3, mildly atypical CBN = 3, severely atypical CBN = 1, and melanoma = 0.

We next examined p16 staining levels within the CBN based on the degree of atypia. p16 intensity and the percentage of positive melanocytes were higher in benign and mildly atypical CBN, and p16 expression decreased as the degree of atypia increased (Figures 1(a) and 1(b), Figure 2). Compared to melanoma, a statistically significant higher percentage of positive melanocytes with p16 was found in CBN which were benign or mildly atypical (69.58 ± 8.23 versus 13.92 ± 6.32, *P* = 0.0002; *N* = 6) and moderately atypical (66.88 ± 12.56 versus 13.92 ± 6.32, *P* = 0.02; *N* = 4), but not in CBN which were severely atypical (20.2 ± 11.96 versus 13.92 ± 6.32; *N* = 5) (Table 2). Similar results were found with p16 staining intensity (Table 2), as well as when comparing atypical CBN to malignant CBN (Table 3). When different categories of atypical CBN were combined together and compared to melanoma and malignant CBN, a statistically significant higher p16 staining intensity and percentage of melanocytes were found with benign/mildly/moderately atypical CBN compared to melanoma and malignant CBN. Also, a statistically significant difference was found when moderately/severely atypical CBN were compared to malignant CBN (Table 3), but not when compared to melanoma (Table 2). Percentage of Ki-67 stained melanocytes was significantly lower in all the CBN categories when compared to melanoma (Figure 1, Table 2).

When p16 expression was compared between the CBN and conventional benign and atypical nevi, no significant difference was found with the benign/mildly atypical CBN and the moderately atypical CBN, but a significant decrease was found with the severely atypical CBN for p16 staining intensity (1.5 ± 0.27 versus 2.69 ± 0.1, *P* = 0.009) and the percentage of melanocytes stained with p16 (20.2 ± 11.96 versus 55.31 ± 8.38, *P* = 0.04) (Figures 1(a) and 1(b), Table 4). There was no significant difference in Ki-67 staining among conventional and CBN for all levels of atypia (Table 4).

## 4. Discussion

We have found that p16 immunohistochemical expression is higher in benign and atypical CBN when compared to melanoma and malignant CBN. This result corresponds to documented higher levels of p16 staining in benign [9–13], congenital [14], conjunctival [15], oral [16], and Spitz nevi [17–19], when compared to melanoma. Our study further subclassified lesions based on the level of atypia, and we found a trend towards decreasing p16 staining as the level of atypia increased. A statistically significant higher level of p16 staining was found between mildly and moderately atypical CBN when compared to melanoma and malignant CBN. Severely atypical CBN, on the other hand, showed levels of p16 staining similar to that of the melanomas examined, and just slightly higher than the malignant CBN, but no statistically significant differences were detected. These findings correspond with the overlapping histologic and biologic features seen between these lesions and melanoma. Similar correlations have been seen between decreasing p16 levels and increasing melanoma tumor thickness [20, 21], increasing stage of disease [22, 23], and metastasis [13, 24, 25].

We also confirmed that Ki-67 is a useful marker in distinguishing atypical CBN from melanoma, as each subtype of CBN had statistically significant lower levels of Ki-67 expression compared to melanoma. Increased p16 levels in the atypical CBN correlated with lower Ki-67 levels, except for the severely atypical CBN (which showed loss of p16, but still relatively low levels of Ki-67). This inverse relationship between Ki-67 and p16 corresponds to studies that have shown that proliferation rate as detected by Ki-67 has been associated with absent or minimal p16 staining in melanomas [7, 13, 26].

Few studies have compared p16 staining in atypical nevi versus benign nevi. Sanki et al. [10] showed that compound nevi expressed p16 more than dysplastic nevi, Keller-Melchior et al. [23] found that p16 was higher in both common and atypical nevi, and George et al. [17] found that the level of dermal p16 staining in atypical Spitz tumors was between that of ordinary Spitz nevi and melanoma. None of these studies, however, subclassified the nevi based on level of atypia. In our study, we found that p16 expression in atypical CBN was similar to that of conventional benign and atypical nevi, except for severely atypical CBN. Subclassification according to degree of atypia in this study has further shown that severely atypical CBN have p16 levels similar to those of melanoma and malignant CBN, and this class of nevi is distinct from mildly and moderately atypical CBN (and conventional nevi).

In conclusion, p16 immunohistochemistry can be helpful to confirm the benign nature of a CBN when the staining is elevated, as seen in conventional, mildly, and moderately atypical CBN. As there are some cases of melanomas which do not lose p16 staining, this marker should not be used singly, but in conjunction with the histologic findings (which remain the gold standard) and other markers such as Ki-67 in making the distinction between atypical cellular blue nevi and melanoma. In interpreting p16 staining, the percentage of melanocytes stained for p16 should be considered more strongly than p16 staining intensity, as our results demonstrated lower *P* values for percentage of melanocytes stained for p16 when CBN were compared to melanoma and malignant CBN. Unfortunately, p16 appears to not be useful in distinguishing severely atypical CBN from malignant CBN and melanoma. As the distinction between severely atypical CBN and malignant CBN is a very difficult diagnostic challenge, these findings are disappointing, but perhaps not surprising, as they likely reflect the overlapping histologic and biologic features between these tumors.

## Figures and Tables

**Table 1:** Patient clinical characteristics, including age, gender, location of tumor, and follow-up data.

Diagnosis	Age	Sex	Location	Clinical follow-up
Benign CBN	21	F	Arm	N/A
Mildly atypical CBN	32	F	Leg	N/A
Mildly atypical CBN	24	M	Arm	N/A
Mildly atypical CBN	63	M	Arm	N/A
Mildly atypical CBN	28	F	Scalp	N/A
Mildly atypical CBN	87	M	Ear	N/A
Moderately atypical CBN	35	M	Scalp	N/A
Moderately atypical CBN	78	F	Face	N/A
Moderately atypical CBN	51	F	Buttock	N/A
Moderately atypical CBN	25	M	Leg	N/A
Severely atypical CBN	49	F	Back	No recurrence after 21 months
Severely atypical CBN	51	F	Leg	No follow-up since diagnosis
Severely atypical CBN	23	M	Chest	Lost to follow-up
Severely atypical CBN	38	F	Buttock	Lost to follow-up
Severely atypical CBN	57	F	Buttock	No follow-up since diagnosis
Superficial spreading melanoma	59	M	Back	No recurrence after 24 months
Superficial spreading melanoma	68	F	Arm	No recurrence after 20 months
Melanoma in transit metastasis	83	F	Arm	Metastatic disease
Spitzoid melanoma	24	F	Chest	No recurrence after 12 months
Melanoma in situ	54	M	Toe	No recurrence after 22 months
Superficial spreading melanoma	32	M	Back	No recurrence after 16 months
Superficial spreading melanoma	60	M	Back	Lost to follow-up
Superficial spreading melanoma	73	M	Neck	No recurrence after 18 months
Nevoid melanoma	56	F	Back	Lost to follow-up
Superficial spreading melanoma	22	F	Leg	No recurrence after 16 months
Spitzoid melanoma	41	M	Back	No recurrence after 22 months
Superficial spreading melanoma	23	F	Arm	No recurrence after 11 months
Malignant CBN	61	F	Foot	No recurrence after 12 months
Malignant CBN	13	M	Face	No follow-up since diagnosis
Malignant CBN	86	M	Back	In transit metastasis
Malignant CBN recurrence	66	M	Face	N/A

**Table 2:** *P* values for comparison between CBN and melanoma for p16 and Ki-67 staining.

CBN versus melanoma (*N* = 12)	p16 intensity	% melanocytes stained with p16	% melanocytes stained with Ki-67
All CBN (*N* = 15)	0.023	0.001	0.019
Benign/mildly atypical CBN (*N* = 6)	0.001	0.0002	0.019
Benign/mildly/moderately atypical CBN (*N* = 10)	0.001	0.000009	0.017
Moderately atypical CBN (*N* = 4)	0.026	0.015	0.015
Moderately/severely atypical CBN (*N* = 9)	0.191	0.061	0.018
Severely atypical CBN (*N* = 5)	0.913	0.658	0.024

**Table 3:** *P* values for comparison between CBN and malignant CBN for p16 staining.

CBN versus malignant CBN (*N* = 4)	p16 intensity	% melanocytes stained with p16
All CBN (*N* = 15)	0.019	0.00008
Benign/mildly atypical CBN (*N* = 6)	0.009	0.0003
Benign/mildly/moderately atypical CBN (*N* = 10)	0.01	0.000002
Moderately atypical CBN (*N* = 4)	0.012	0.014
Moderately/severely atypical CBN (*N* = 9)	0.047	0.017
Severely atypical CBN (*N* = 5)	0.234	0.308

**Table 4:** *P* values for comparison between CBN and conventional benign/atypical melanocytic nevi for p16 and Ki-67 staining.

CBN versus conventional benign/atypical melanocytic nevi (*N* = 8)	p16 intensity	% melanocytes stained with p16	% melanocytes stained with Ki-67
All CBN (*N* = 15)	0.036	0.808	0.318
Benign/mildly atypical CBN (*N* = 6)	0.728	0.248	0.37
Benign/mildly/moderately atypical CBN (*N* = 10)	0.516	0.237	0.45
Moderately atypical CBN (*N* = 4)	0.576	0.474	0.795
Moderately/severely atypical CBN (*N* = 9)	0.022	0.331	0.438
Severely atypical CBN (*N* = 5)	0.009	0.044	0.428
